# Ventricular fibrillation count by noise oversensing in a cardiac resynchronization therapy‐defibrillator induced by side branch protection during percutaneous coronary intervention

**DOI:** 10.1002/joa3.12749

**Published:** 2022-07-07

**Authors:** Sho Tanabe, Masao Takahashi, Takashi Kimura, Rintaro Hojo, Seiji Fukamizu

**Affiliations:** ^1^ Department of Cardiology Tokyo Metropolitan Hiroo Hospital Tokyo Japan

**Keywords:** cardiac resynchronization therapy, defibrillators, noise oversensing, percutaneous coronary intervention, ventricular fibrillation

An 80‐year‐old man was admitted to the Department of Cardiology at our institution for treatment of silent myocardial ischemia. The patient had left ventricular systolic dysfunction(left ventricular ejection fraction: 39%), brady‐tachycardia syndrome, advanced atrioventricular block, and prior ventricular fibrillation. Therefore, a cardiac resynchronization therapy‐defibrillator (CRT‐D) (Claria MRI Quad CRT‐D Surescan System, Medtronic) had already been implanted as a means of secondary prevention. Coronary angiography performed after a diagnosis of congestive heart failure showed 90% stenosis of the mid‐left anterior descending artery (LAD) and 75% stenosis of the diagonal branch (Medina type 1, 0, 1). On admission, percutaneous coronary intervention (PCI) was performed for significant stenosis.

During PCI, the first guidewire (SION™, ASAHI INTEC) was advanced through the LAD lesion until it reached the ventricular apex, and the second guidewire (SION blue™, ASAHI INTEC) was advanced through the diagonal branch (Figure 1). The electrocardiogram (ECG) monitor showed sudden, repeated, long pauses when the guidewire was inserted into the diagonal branch (Figure 2). Therefore, we removed the guidewire immediately, and the ECG showed a return to biventricular pacing. CRT‐D interrogation, when the second guidewire was inserted into the diagonal branch again, revealed ventricular fibrillation (VF) counts, detected by noise oversensing, and pacing inhibition was caused by the VF counts (Figure 3). The noise signal was a discrete potential. Therefore, we considered a relationship between noise oversensing and guidewire insertion or diagonal branch stenosis. Hence, we changed the pacing mode from DDD to DOO, and the antiarrhythmics (therapy for tachycardia control) were suspended. Subsequently, we performed balloon inflation of the stenosis of the LAD and the diagonal branch to correct ischemia. After balloon dilation, noise oversensing of the right ventricular (RV) lead was not observed when the same guidewire was reinserted into the diagonal branch. We performed the entire procedure using intravascular ultrasound (IVUS) and successfully completed it without any further complications. Finally, complete device interrogation was performed, which showed optimal sensing and pacing thresholds in atrial and ventricular leads. Additionally, there was no evidence of lead fracture or noise because of changes in the position or arm movement. The patient was discharged 2 days after the procedure and was followed up at our outpatient department without any recurrence of heart failure.

**FIGURE 1:**
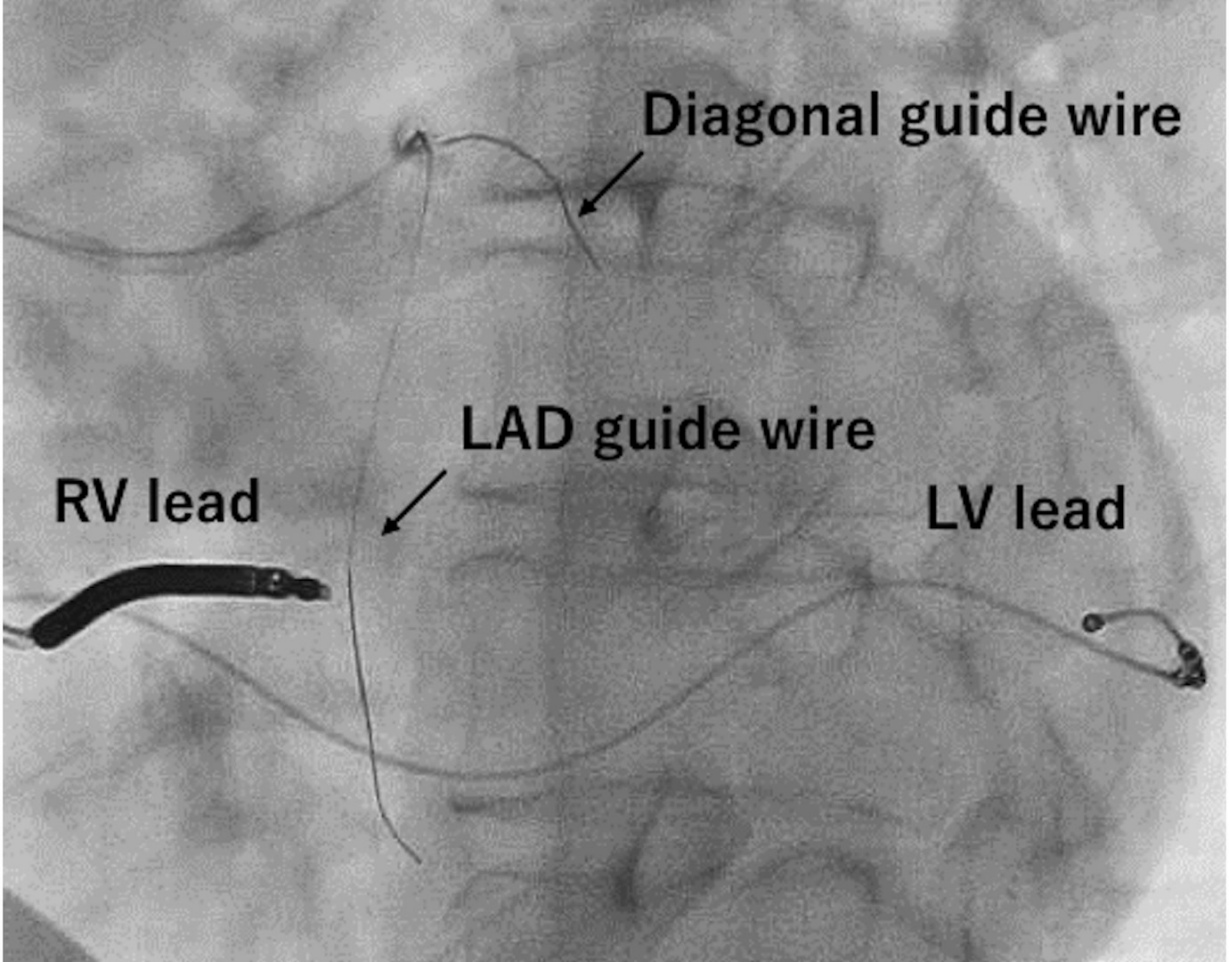
Ventricular leads were located away from the diagonal branch guide wire

**FIGURE 2:**
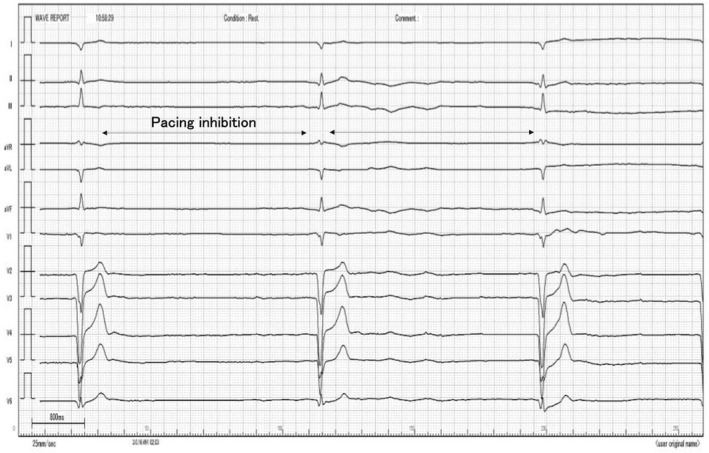
Long pause because of pacing inhibition occurred in electrocardiogram during the insertion of second guidewire into diagonal branch.

**FIGURE 3:**
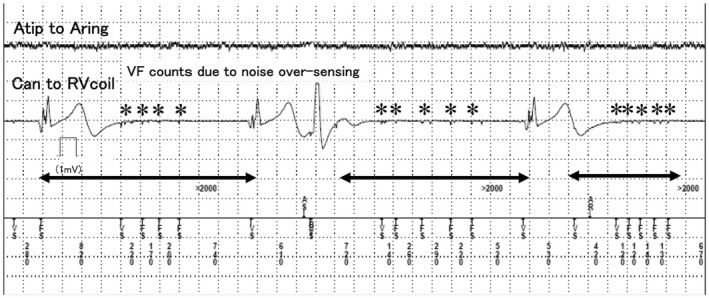
The channels recorded from top to bottom: The atrial tip to the atrial ring and the can to the right ventricular coil. Asterisks showed noise detection on right ventricular lead. Arrows showed a long pause because of noise oversensing.

In this paper, we consider two important findings for the elucidation of noise oversensing in our case. One is the intermittent discrete potential of noise signals in the RV lead, and the other is the wire bias of the two guidewires within the coronary stenosis. From this intermittent discrete potential and circumstantial evidence, myopotential, lead fraction, and external electromagnetic interference are unlikely. Previous reports have described inappropriate shock delivery because of noise oversensing in an implantable cardioverter‐defibrillator device during PCI.^1^, ^2^ A case report^1^ demonstrated that LAD guidewires had actual contact with the helix tip of RV leads, which were screwed into the right mid‐anterior septum, and the intermittent discrete noise signals might be caused by the wire‐helix mechanical interaction. However, noise oversensing in our case occurred during the insertion of the second guidewire into the diagonal branch, which was far from the RV lead helix tip. Therefore, we focused on IVUS imaging findings (Figure 4). It was found that the two guidewires in the LAD stenosis were fairly close to each other and that the inner diameter of the LAD and diagonal branch expanded after balloon dilation, which increased the degree of freedom of the two guidewires. We speculated that mechanical interference between the two guidewires in the stenosis was conducted through the first guide wire and sensed by the helix tip of the RV lead as intermittent discrete potentials, and the amplitude of the potentials of this mechanical interference was lower than the sensing threshold after stenosis release by balloon dilation, which reduced the wire bias.

**FIGURE 4:**
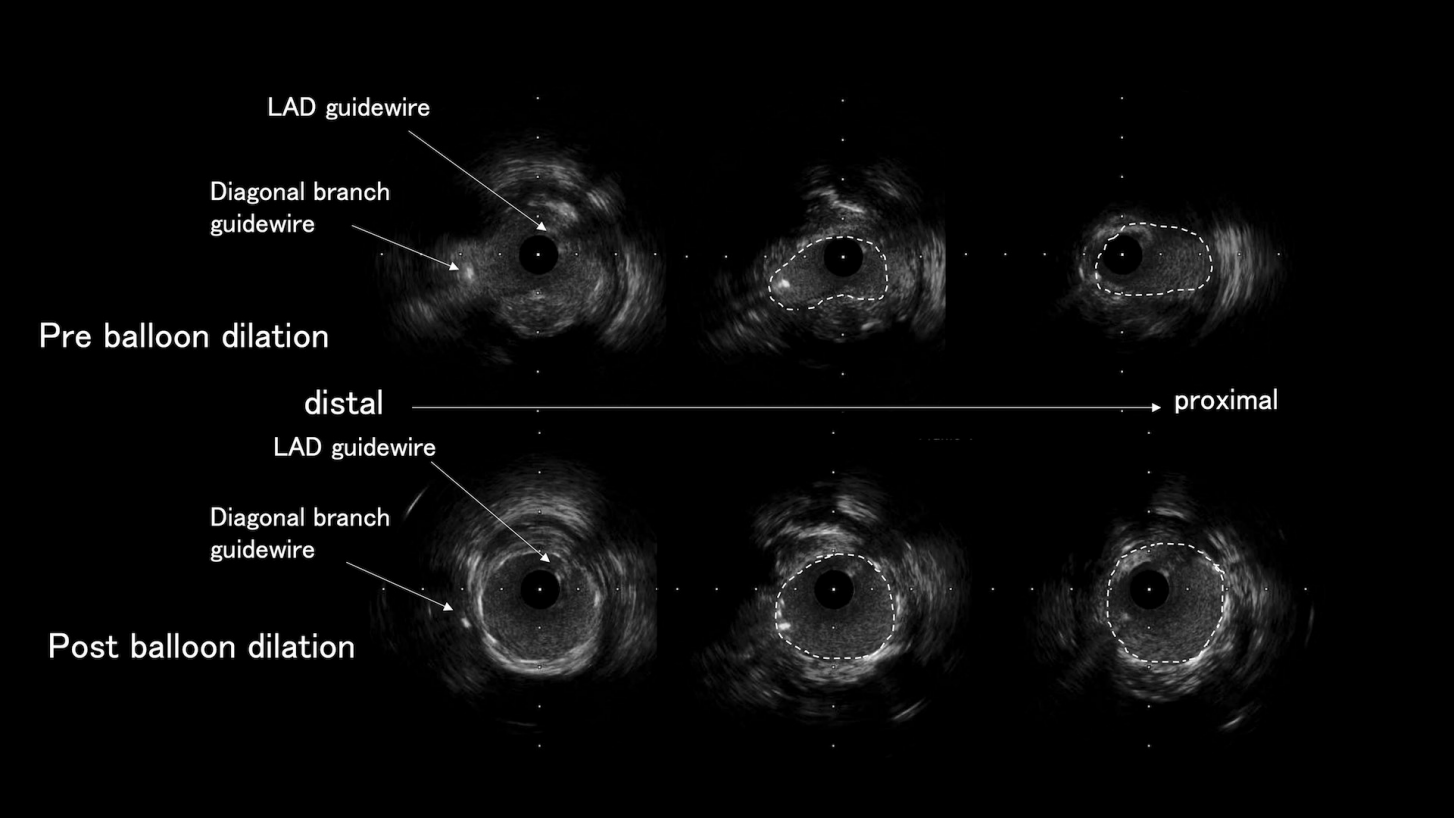
Imaging of intravascular ultrasound at pre balloon dilation (upper side) revealed that two guidewires in stenosis were very close to each other. Imaging of intravascular ultrasound at post balloon dilation (bottom side) indicated that the release of stenosis reduced the wire bias.

We report the case of a patient with a CRT‐D who underwent PCI and was noted to have a long pause because of noise oversensing induced by the side branch protection located on the diagonal branch. Cardiologists should consider the possibility of PCI‐related “noise” regardless of the distance between the guidewire and lead position. Patients should be carefully observed during PCI to avoid pacing inhibition or delivery of inappropriate shocks in patients with cardiac implantable electronic devices.

## APPROVAL OF THE RESEARCH PROTOCOL

All procedures performed in our case involving human participants were in accordance with the ethical standards of the Institutional and National Research Committee and the 1964 Helsinki Declaration and its later amendments or comparable ethical standards.

## INFORMED CONSENT

Written informed consent was obtained from the patient for the publication of this case report and accompanying images.

## REGISTRY AND THE REGISTRATION NO.

N/A.

## ANIMAL STUDIES

N/A.

## CONFLICT OF INTEREST

None declared.

## ETHICAL APPROVAL

All procedures performed in our case involving human participants were in accordance with the ethical standards of the institutional and national research committee and the 1964 Helsinki Declaration and its later amendments or comparable ethical standards.

## PATIENT CONSENT STATEMENT

Written informed consent was obtained from the patient for the publication of this case report and accompanying images.

## PERMISSION TO REPRODUCE MATERIAL FROM OTHER SOURCES

No third‐party materials or sources were reproduced in the current study.

## CLINICAL TRIAL REGISTRATION

Not applicable for the current study.
